# Unemployment, Employability and COVID19: How the Global Socioeconomic Shock Challenged Negative Perceptions Toward the Less Fortunate in the Australian Context

**DOI:** 10.3389/fpsyg.2020.594837

**Published:** 2020-10-15

**Authors:** Aino Suomi, Timothy P. Schofield, Peter Butterworth

**Affiliations:** ^1^Research School of Population Health, The Australian National University, Canberra, ACT, Australia; ^2^Institute of Child Protection Studies, The Australian Catholic University, Melbourne, VIC, Australia; ^3^Melbourne Institute of Applied Economic and Social Research, The University of Melbourne, Melbourne, VIC, Australia

**Keywords:** COVID19, employability, personality, Big Five, public policy, unemployment

## Abstract

Unemployed benefit recipients are stigmatized and generally perceived negatively in terms of their personality characteristics and employability. The COVID19 economic shock led to rapid public policy responses across the globe to lessen the impact of mass unemployment, potentially shifting community perceptions of individuals who are out of work and rely on government income support. We used a repeated cross-sections design to study change in stigma tied to unemployment and benefit receipt in a pre-existing pre-COVID19 sample (*n* = 260) and a sample collected during COVID19 pandemic (*n* = 670) by using a vignette-based experiment. Participants rated attributes of characters who were described as being employed, working poor, unemployed or receiving unemployment benefits. The results show that compared to employed characters, unemployed characters were rated substantially less favorably at both time points on their employability and personality traits. The difference in perceptions of the employed and unemployed was, however, attenuated during COVID19 with benefit recipients perceived as more employable and more Conscientious than pre-pandemic. These results add to knowledge about the determinants of welfare stigma highlighting the impact of the global economic and health crisis on perception of others.

## Introduction

The onset of COVID19 pandemic saw unemployment climb to the highest rate since the Great Depression in many regions globally^1^. Over just one month, from March to April 2020 unemployment rate in the United States increased from 4.4% to over 14.7% and in Australia the effective rate of unemployment increased from 5.4 to 11.7% (Australian Bureau of Statistics, 2020)^2^. In Australia, a number of economic responses were rapidly introduced including a wage subsidy scheme (Jobkeeper) to enable employees to keep their employees connected to the workforce, one-off payments to many welfare recipients, and a doubling of the usual rate of the unemployment benefits (Jobseeker payment) through a new Coronavirus supplement payment. At the time of writing in July 2020, many countries, including Australia remain in the depths of a health and economic crisis.

A rich research literature from a range of disciplines has documented the pervasive negative community views toward those who are unemployed and receiving unemployment benefits, with the extent of this “welfare stigma” being particularly pronounced in countries with highly targeted benefit systems such as the United States and Australia (Fiske et al., 2002; Baumberg, 2012; Contini and Richiardi, 2012; Schofield and Butterworth, 2015). The stigma and potential discrimination associated with unemployment and benefit receipt are known to have negative impacts on health, employability and equality (for meta-analyses, see Shahidi et al., 2016). In addition, the receipt of unemployment benefits co-occurs with other stigmatized characteristics such as poverty and unemployment (Schofield and Butterworth, 2018a). The changing context related to the COVID19 crisis provides a novel opportunity to better understand the determinants of stigmatizing perceptions of unemployment and benefit receipt.

Negative community attitudes and perceptions of benefit recipients are commonly explained by the concept of “deservingness” (van Oorschot and Roosma, 2017). The unemployed are typically seen as less deserving of government support than other groups because they are more likely to be seen as responsible for their own plight, ungrateful for support, not in genuine need (Petersen et al., 2011; van Oorschot and Roosma, 2017), and lacking reciprocity (i.e., seen as taking more than they have given – or will give – back to society; van Oorschot, 2000; Larsen, 2008; Petersen et al., 2011; Aarøe and Petersen, 2014). Given the economic shock associated with COVID19, unemployment and reliance on income support are less likely to seen as an outcome within the individuals control and may therefore amplify perceptions of deservingness. Prior work has shown that experimentally manipulating perceived control over circumstances does indeed change negative stereotypes (Aarøe and Petersen, 2014).

A number of experimental paradigms have been used to investigate perceptions of “welfare recipients” and the “unemployed.” The stereotype content model (SCM; Fiske et al., 2002), for example, represents the stereotypes of social groups on two dimensions: warmth, relating to being friendly and well–intentioned (rather than ill–intentioned); and competence, relating to one’s capacity to pursue intentions (Fiske et al., 2002). Using this model, the “unemployed” have been evaluated as low in warmth and competence across a variety of welfare regime types (Fiske et al., 2002; Bye et al., 2014). The structure of stereotypes has also been studied using the Big Five personality dimensions (Schofield and Butterworth, 2018b; Schofield et al., 2019): Openness, Conscientiousness, Extraversion, Agreeableness, and Emotional Stability (for background on the Big Five see: Goldberg, 1993; Hogan et al., 1996; Saucier and Goldberg, 1996; McCrae and Terracciano, 2005; Srivastava, 2010; Chan et al., 2012; Löckenhoff et al., 2014). There are parallels between the Big Five and the SCM: warmth relating to the dimension of Agreeableness, and competence relating to Conscientiousness (Digman, 1997; Ward et al., 2006; Cuddy et al., 2008; Abele et al., 2016) and these constructs have been found to predict employability and career success (Barrick et al., 2001; Cuesta and Budría, 2017). Warmth and agreeableness have also been linked to the welfare-specific characteristics of deservingness (Aarøe and Petersen, 2014).

The term “employability” has been previously defined as a set of achievements, skills and personal attributes that make a person more likely to gain employment and leading to success in their chosen career pathway (Pegg et al., 2012; O’Leary, 2017, 2019). While there are few studies examining perceptions of others, perceptions of one’s own employability have been recently studied in university students, jobseekers (Atitsogbe et al., 2019) and currently employed workers (Plomp et al., 2019; Yeves et al., 2019), consistently showing higher levels of perceived employability being linked to personal and job-related wellbeing as well as career success. Examining other’s perceptions of employability may be more relevant to understand factors impacting on actual employment outcomes. A majority of studies examining other’s perceptions of employability have focused on job specific skills study (Lowden et al., 2011; Dhiman, 2012; Saad and Majid, 2014).

Building on this previous work, our own research has focused on the effects of unemployment by drawing on frameworks of Big Five, SCM and employability in pre-COVID19 samples (Schofield and Butterworth, 2018b; Schofield et al., 2019). Our studies consistently show that unemployed individuals receiving government payments are perceived as less employable (poorer “quality” workers and less desirable for employment) and less Conscientious. We found similar but weaker pattern related to Agreeableness, Emotional Stability, and the extent that a person is perceived as “uniquely human” (Schofield et al., 2019). Further, we found that vignette characters described as currently employed but with a history of welfare receipt were indistinguishable from those described as employed and with no reference to benefit receipt (Schofield et al., 2019). Findings such as this provide experimental evidence that welfare stigma is malleable and can be challenged by information inconsistent with negative stereotype (Schofield and Butterworth, 2018b; Schofield et al., 2019; see also Petersen et al., 2011).

The broad aim of the current study was to extend this previous work by examining the impact of COVID19 on person perceptions tied to employment and benefit recipient status. It repeats a pre-COVID19 study of an Australian general population sample in the COVID19 context, drawing on the same sampling frame, materials and study design to maximize comparability. The study design recognizes that the negative perceptions of benefit recipients may reflect a combination of difference sources of stigma: poverty, lack of work, and benefit receipt. Therefore, the original study used four different conditions to seek to differentiate these different sources: (1) *Employed*; (2) *Working poor*; (3) *Unemployed*; and (4) *Unemployed benefit recipient*. Finally, for the COVID19 sample we added a novel fifth condition: (5) *Unemployment benefit recipient also receiving the “Coronavirus” supplement*. We except that the reference to a payment specifically applicable to the COVID19 context may lead to more favorable perceptions (more deserving) than the other unemployed and benefit receipt characters.

The study capitalizes on a major exogenous event, the COVID19 crisis, which we hypothesize will alter perceptions of deservingness by fundamentally challenging social identities and perceptions of one’s own vulnerability to unemployment. The study tests three hypotheses, and in doing so makes an important empirical and theoretical contribution to understanding how deservingness influences person perception, and understanding of the potential “real world” barriers experienced by people seeking employment in the COVID19 context.

### Hypothesis 1

The pre-COVID19 assessment uses a subset of data from a pre-registered study, but this reuse of the data was not preregistered^3^. We hypothesize that, at Time 1 (pre-COVID19 assessment) we will find that employed characters will be rated more favorably than characters described as unemployed and receiving unemployment benefits, particularly on dimensions of Conscientiousness, Worker and Boss suitability. Moreover, we expect a gradient in perceptions across the four experimental conditions, from employed to working poor, to unemployed to unemployed receiving benefits and to show a similar trend for the other outcome measures included in the study.

### Hypothesis 2

We hypothesize that the character in the unemployed condition(s) would be rated less negatively relative to the employed condition(s) at Time 2, compared to Time 1. We predict a two-way interaction between time and condition for the key measures (Conscientiousness, Worker and Boss suitability) and a similar trend on other outcomes.

### Hypothesis 3

We expect that explicit reference to the unemployed benefit character receiving the “Coronavirus supplement” payment will increase the salience of the COVID19 context and lead to more positive ratings of this character relative to the standard unemployed benefit condition in the pre-COVID19 and COVID19 occasions.

## Materials and Methods

### Participants

Two general population samples (pre-COVID19 and COVID19) were recruited from the same source: The Australian Online Research Unit (ORU) panel. The ORU is an online survey platform that provides access to a cohort of members of the general public who are interested in contributing to research. The ORU randomly selects potential participants who meet study eligibility criteria, and provides the participant with an incentive for their participation. The sample for the Time 1 (pre-COVID19) occasion was part of a larger study (768 participants) collected in November 2018. From this initial dataset, we were able to use data from 260 (50.1% female, *M* Age = 42.1 [16.7] years, range: 18–82) participants who were presented with the one vignette scenario that we could replicate at the time of the social restrictions applicable in the COVID19 context (i.e., the vignette character was not described as going out and visiting friends, as these behaviors were illegal at Time 2). The sample for Time 2 (COVID19) was collected in May–June 2020, at the height of the lock down measures in Australia and included 670 participants (40.5% female, *M* Age = 51.0 [15.8] years, range: 18–85). The two samples were broadly similar (see below), though the proportion of male participants at Time 2 was greater than at Time 1.

### Sampling

The pre-COVID assessment at Time 1 was restricted to those participants who completed the social-distancing consistent vignette in the first place to avoid potential order/context effects. This provided, on average, 65 respondents in each of the four experimental conditions. Using the results from our previous published studies as indicators of effect size (Schofield and Butterworth, 2018b; Schofield et al., 2019). Monte Carlo simulation was used to identify the Time 2 sample size that would provide 90% power to detect an interaction effect that represented a 50% decline in the difference between the two employment and two unemployment conditions on the three-key measures at the COVID occasion relative to the pre-COVID difference. This sample size of 135 per condition also provided between 60 and 90% power to detect a difference of a similar magnitude between the employed and unemployment benefit conditions across the two measurement occasions. Given previous evidence that the differences between employed and unemployed/welfare conditions is robust and large for Conscientiousness and Worker suitability (Schofield and Butterworth, 2018b), the current study is also adequately powered to detect the most replicable effects of unemployment and welfare on perceptions of a person’s character (even in the absence of the hypothesized interaction effect).

### Materials and Procedure

The procedures were identical on both study occasions. Participants read a brief vignette that described a fictional character, and then rated the character on measures reflecting personality dimensions, their suitability as a worker or boss, morality, warmth, and competence, and the participant’s beliefs the character should feel guilt and shame, or feel angry and disgusted. At Time 1 (pre-COVID19 context) participants then repeated this process with a second vignette, but we do not consider data from the second vignette.

#### Manipulation

The key experimental conditions were operationalized by a single sentence embedded within the vignette that was randomly allocated to different participants (employed: “S/he is currently working as a sales assistant in a large department store”; working poor: “S/he is currently working as a sales assistant, on a minimum-wage, in a large department store”; unemployed: “S/he is currently unemployed”; and receipt of unemployment benefits: “S/he is currently unemployed, and is receiving government benefits due to his/her unemployment”). The four experimental conditions were identical at both time points. At Time 2, an additional COVID19-specific condition was included (to maximize the salience of the COVID19 context): “S/he is currently unemployed and is receiving government benefits, including the Coronavirus supplement, due to his/her unemployment.”

All three study conditions will imply poverty/low income. In Australia, few minimum-wage jobs are supplemented by tips, and so a minimum-wage job indicates a level of relative poverty. A full-time worker in a minimum wage job is in the bottom quartile of income earners (Australian Bureau of Statistics, 2017). Prior to the COVID19 crisis and the increase in payment level, a single person with no dependents receiving unemployment benefits received approximately 75% of the minimum-wage in cash assistance. During COVID19 and at the time of the data collection, the rate of pay exceeds the minimum-wage.

Several characteristics of the vignette character, including age and relationship status, were balanced across study participants. Age was specified as either 27 or 35 years, relationship status was either “single” or “lives with his/her partner.” The character’s gender was also varied and names were stereotypically White.

### Design

**FIGURE 1:**
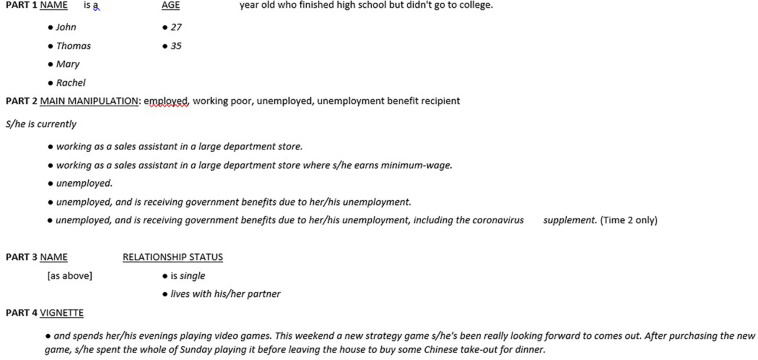
Outline of vignette construction in 4 parts. Bullet pointed options replace the underlined text, with gendered pronouns in each option selected to match character name.

For Time 1, manipulated characteristics yielded 32 unique vignettes, comprised of four key experimental conditions (employed, working poor, unemployed, and unemployment benefits) × 2 ages × 2 genders × 2 relationship statuses. For Time 2, manipulated characteristics yielded 40 unique vignettes, comprised of five key experimental conditions (employed, working poor, unemployed, unemployment benefits, and unemployed + coronavirus supplement) × 2 ages × 2 genders × 2 relationship statuses. The *vignette template* construction is presented in Figure 1 including each component of the vignette that was randomly varied.

### Comprehension Checks

In both studies, participants were required to affirm consent after debriefing or had their data deleted. Participant comprehension of the vignettes was checked via three free-response comprehension questions about the character’s age and weekend activities. Participants who did not answer any questions correctly were not able to continue the study.

### Outcome Measures

Personality, employability (suitability as a worker or boss), communion and agency, cognitive and emotional moral judgments, and dehumanization were included as the study outcomes. While not all personality or character dimension measures can be considered as negative or positive, higher scores were used in the study to indicate more “favorable” perceptions by the participants of the characters.

#### Personality

The Ten Item Personality Inventory was used to measure the Big Five (Gosling et al., 2003) and adapted to other–oriented wording (i.e., “I felt like the person in the story was…”) (Schofield et al., 2019). Two items measured each trait via two paired attributes. One item contained positive attributes and one contained negative attributes. Participants indicated the extent to which “I think [Name] is [attributes]” from 1 (strongly disagree) to 7 (strongly agree). The order of these 10 items was randomized. Agreeableness (α = 0.54) was assessed from “sympathetic, warm” and “critical, quarrelsome” (reversed); Extraversion (α = 0.50) was assessed from “extraverted, enthusiastic” and “reserved, quiet” (reversed); Conscientiousness (α = 0.76) was assessed from “dependable, self-disciplined” and “disorganized, careless” (reversed); Openness to experience (α = 0.36) was assessed from “open to new experiences, complex” and “conventional, uncreative” (reversed); Emotional stability (α = 0.65) was assessed from “calm, emotionally stable.” and “anxious, easily upset” (reversed). The order of these 10 items was randomized.

#### Employability

Single item measures: “I think [Name] would be a good worker” (*Worker suitability*) and “I think [Name] would be a good boss” (*Boss suitability*) were rated on the same scale as the personality measure. The order of these two items was randomized. Higher scores indicated better employability.

#### Communion and Agency

Communion and agency was assessed using Bocian et al. (2018) adaptation of Abele et al. (2016) scale that measures the fundamental dimensions of communion and agency using two-subscales for each dimension. The morality and warmth subscales are seen as measures of communion (referred to as warmth in SCM; Fiske, 2018); while the competence and assertiveness subscales measure agency (what Fiske refers to as competence in SCM; Fiske, 2018). This subscale structure has been identified in multiple samples. Participants indicated the extent to which “I think [Name] [attributes]” from 1 (not at all) to 5 (very much so). Morality (α = 0.92) was measured with six items, e.g., “is just,” “is fair”; Warmth (α = 0.96) with six items, e.g., “is caring,” “is empathetic”; Competence (α = 0.90) with five items, e.g., “is efficient,” “is capable”; and Assertiveness (α = 0.83) with six items, e.g., “is self-confident,” “stands up well under pressure.” These items were presented in a random order.

#### Dehumanization

Dehumanization was measured with a composite scale of two-items drawn from Bastian et al. (2013). Based on prior research, we measured dehumanization with two items: “I think [Name] is mechanical and cold, like a robot” and “I think [Name] lacked self-restraint, like an animal” order of these two items was randomized. We reverse coded the two items for the analyses for consistency for the other variables, so that higher scores were indicative of more favorable perceptions.

#### Moral Emotions

Moral emotions were measured by four items that asked about emotional responses to the character that were framed as self-condemning or other-condemning (Haidt, 2003; Giner-Sorolla and Espinosa, 2011). Two other-condemning items asked the participant about their own emotional response to the character in the vignette (Anger: “[Name]’s behavior makes me angry”; Disgust: “I think [Name] is someone who makes me feel disgusted,” α = 0.92). The two self-condemning items asked about the character’s emotional response (Guilt: “[Name] should feel guilty about [his/her] behavior”; Shame: “I think [Name] should feel ashamed of [him/her]self”; α = 0.95). We reverse coded the two scales to ensure consistency with other variables, with higher scores indicative of more favorable perceptions.

### Analytical Strategy

With the exception of the Moral emotion (and Communion and Agency) scales that are new to this study and the previously tested Openness to Experience, our previous research has demonstrated differences between the ratings of employed and unemployed characters on the included outcome measures (Schofield and Butterworth, 2018b; Schofield et al., 2019). We undertake the analysis using a four-step process. We use mixed-effects multi-level models, with the 14 outcome measures nested within participants, and predicted by fixed (between-person) terms representing the experimental “Condition,” “Time” (pre-/COVID19) and their interaction, and controlling for measure differences and allowing for random effects at the participant level: i) We initially assessed the effect of condition in the pre-COVID19 occasion to establish the baseline pattern of results; ii) we then evaluated the interaction term and, specifically, the extent to which the baseline difference observed between employment and unemployment conditions is attenuated at Time 2 (COVID19 occasion); iii) we tested the three-way interaction between condition, occasion and measure to assess whether this two-way interaction varies across the outcome measures; and if significant iv) repeated the modeling approach using separate linear regression models for each outcome measure. Our initial model contrasts the two employed (employed and working poor) and unemployed (unemployed and benefit receipt) conditions. The second model examines the four separate vignette conditions separately, differentiating between unemployed and unemployed benefit conditions. Finally, we contrast the three unemployment benefit conditions: (1) unemployment benefit recipients at Time 1; (2) unemployment benefit recipients at Time 2; and (3) unemployment benefit recipients receiving the Coronavirus payment at Time 2. For all models, we consider unadjusted and adjusted results (controlling for participant demographics). To address a potential bias from gender differences between samples, post-stratification weights were calculated for the COVID19 sample to reflecting the gender by age distribution of the pre-COVID19 sample. All models were weighted.

## Results

The two samples from Time 1 (pre-COVID19) and Time 2 (COVID19) were comparable on all demographic variables, except for gender (χ^2^ [1, 923] = 7.04, *p* < 0.001) and employment (χ^2^ [1, 910] = 27.66, *p* < 0.001): The gender distribution was more balanced at Time 1 with 49.8% of males, compared to 59.5% of males at Time 2. There was also a significant increase in unemployment with 20.9% of Time 1 participants out of work compared to 39.3% of the Time 2 participants. This was likely reflective of the employment rate nearly doubling in Australia during COVID19 crisis. Bivariate correlations showed significant positive correlations between all 14 outcomes (*p*’s < 0.001), except for Extraversion that was only positively correlated with Emotional Stability, boss suitability, warmth, assertiveness, and competence (*p’*s < 0.05).

### Contrasting Employed and Unemployed Characters

The results, both adjusted and unadjusted, from the initial overall multilevel model using a binary indicator of whether vignette characters were employed (those in the employed or working poor conditions) or unemployed (unemployed or welfare) and testing the interaction between vignette Condition and Time (pre-COVID19 vs COVID19) are presented in the Supplementary Table S1. The adjusted results (holding participant age, gender, employment, and education constant) indicated that the unemployed characters were rated lower than the employed characters at Time 1 (*b* = −0.57). This difference in the ratings of employed and unemployed characters was reduced in the COVID19 assessment at Time 2, declining from 0.57 to 0.26, across all the outcome measures. The addition of the three-way interaction between Condition, Time and outcome measure significantly improved overall model fit, χ^2^ (52) = 482.94, *p* < 0.001, indicating the interaction between Condition and Time varied over measures.

A series of separate regression models considering each outcome separately (see Supplementary Table S2) showed a significant effect of Condition (employment rated higher than unemployment) at Time 1 (pre-COVID) for all outcomes except Openness and Extraversion. The lower ratings for unemployed relative to employed characters were significantly moderated at Time 2 on the Competence, Worker and Boss suitability, and Guilt/Shame outcomes (*p’*s < 0.05).

### COVID19 and Perceptions of Unemployment Benefit Recipients

The next set of analyses consider the four separate vignette conditions, differentiating between the unemployed and unemployed benefit recipient conditions. The overall mixed-effects multilevel model incorporating the four distinct vignette conditions provided evidence of significant effects for Condition and Condition by Time in both adjusted and unadjusted models. The result for the adjusted model (Table 1), averaged across the various outcomes, replicated the previous finding of a difference in ratings of employed and unemployed characters at Time 1 (pre-COVID19): relative to the employed condition, there was no difference in ratings of the working poor, but the unemployed and the unemployed benefit recipient characters were rated less favorably. There was some evidence of a gradient across the unemployed characters: the average rating of the unemployed condition was higher than the unemployed benefit condition, though this difference was not statistically significant. In the presence of the interaction effect, the non-significant effect of Time shows that, averaged across all the outcome measures, there was no difference in the rating of the characters in the employed condition on the pre-COVID19 and COVID19 occasions. We tested for the effect of sociodemographic characteristics as covariates in the adjusted models (employment and benefit receipt status, education, age, and gender) but found no main effects of any of the covariates except for gender: females tended to rate characters higher (*b* = 0.13, 95% CI [0.04, 0.21]) compared to males. Testing the heterogeneity of these patterns across outcomes via the inclusion of a three-way interaction between vignette condition, occasion and measure significantly improved overall model fit, χ^2^ (104) = 533.40, *p* < 0.001, prompting analysis of each outcome separately.

**TABLE 1:** Adjusted fixed effects estimates of outcomes as a function of interactions between condition and time.

	Coeff.	SE (robust)	*z*	*p*	[95% CI]	
**Time (ref Time 1)**						
Time 2	–0.06	0.09	–0.73	0.47	–0.24	0.10
**Condition (ref E)**						
WP	0.02	0.10	0.18	0.86	–0.19	0.22
UE	–0.50	0.11	–4.70	<0.001	–0.71	–0.29
UB	–0.61	0.11	–5.84	<0.001	–0.81	–0.41
**Time × Condition**						
Time 2 WP	0.01	0.12	0.11	0.91	–0.23	0.26
Time 2 UE	0.22	0.13	1.74	0.08	–0.03	0.47
Time 2 UB	0.33	0.13	2.51	0.01	0.07	0.58

*Conditions: E, employed; WP, working poor; UE, unemployed; UB, unemployment benefits.*

**FIGURE 2:**
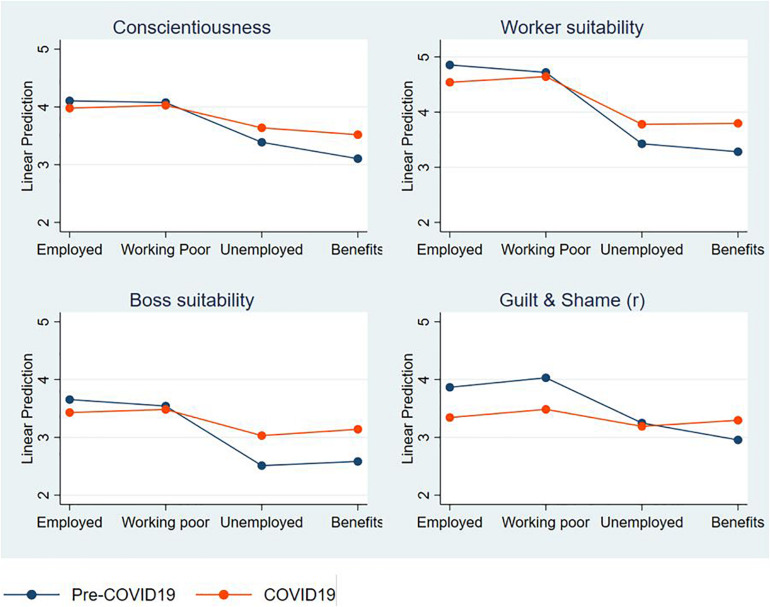
Interaction effect of Time (COVID19) by Condition marginal mean ratings on four outcomes: Conscientiousness, Worker suitability, Boss suitability, and Guilt and Shame (reversed).

The separate linear regressions for each outcome measure (Supplementary Table S3) show that ratings of unemployed benefit recipients at the Time 1 (pre-COVID19) were significantly lower than the employed characters for all outcomes except Openness and Extraversion. Statistically significant Condition by Time terms indicated that the unemployed benefit effect was moderated at Time 2 (COVID19) for the three key outcome measures identified in previous research (Conscientiousness, Worker and Boss suitability) and for the measure of Guilt and Shame. Figure 2 depicts this interaction for these four outcomes. These occurred in two profiles. For Conscientiousness, Worker and Boss suitability, COVID19 attenuated the negative perceptions of unemployed relative to employed characters, providing support for Hypothesis 2. By contrast, COVID19 has induced a new difference, such that participants thought employed characters should feel higher levels Guilt and Shame at Time 2, compared to Time 1. While the “working poor” condition was not central to the COVID19 hypotheses, we note that we found no evidence that ratings of these characters on any outcome differed from the standard employed character, or that this difference was changed in assessment at Time 2 (COVID19 occasion).

### The Impact of COVID19 on Perceptions of Unemployment Benefit-Recipients

The inclusion of the fifth COVID19-specific unemployment benefit condition did not generate more positive (or different) ratings than the standard unemployment benefit condition. Overall mixed-effects multilevel models, both adjusted and unadjusted, indicated that participants in the Coronavirus supplement condition (adjusted model: *b* = 0.26, 95% CI [0.06, 0.45]) and the general unemployed benefit recipient condition at Time 2 (adjusted model: *b* = 0.28, 95% CI [0.08, 0.48]) were both rated more favorably in comparison to unemployed benefit recipients at Time 1. There was no difference between these two Time 2 benefit recipient groups (*b* = 0.03, 95% CI [−0.12, 0.19]). These results did not support hypothesis 3.

## Discussion

Previous research has demonstrated that people who are unemployed, and particularly those receiving unemployment benefits, are perceived more negatively and less employable than those who are employed. However, the economic shock associated with the COVID19 crisis is likely to have challenged people’s sense of their own vulnerability and risk of unemployment, and altered their perceptions of those who are unemployed and receiving government support. The broad aim of the current study was to examine the potential effect of this crisis on person perceptions tied to employment and benefit recipient status. We did this by presenting brief vignettes describing fictional characters, manipulating key experimental conditions related to employment status, and asking study participants to rate the characters’ personality and capability. We contrasted results from two cross-sectional general population samples collected before and during the COVID19 crisis.

The pre-COVID19 assessment replicated our previous findings (e.g., Schofield and Butterworth, 2018b) showing that employed characters are perceived more favorably than those who were unemployed and receiving government benefits on measures of Conscientiousness and suitability as a worker. These findings supported Hypothesis 1. In comparison, the assessment conducted during the COVID19 crisis showed that unemployed and employed characters were viewed more similarly on these same key measures, with a significant interaction effect providing support for Hypothesis 2. Our third hypothesis, suggesting that n reference to the Coronavirus Supplement (an additional form of income support introduced during the pandemic) would enhance ratings of unemployed benefit recipients at the second assessment occasion, was not supported. We found that benefit recipients at Time 2 were rated more favorably than the benefit group at Time 1, irrespective of whether this COVID19-specific payment was referenced. This suggests the broader context in which the study was conducted was responsible for the change in perceptions.

We sampled participants from the same population, used identical experimental procedures, and found no difference over time in the ratings of employed characters on the key outcome measures of employability (Worker and Boss suitability) and Conscientiousness. The more favorable ratings of unemployed and benefit receiving characters at Time 2 is likely to reflect how the exogenous economic shock brought about by the COVID19 crisis challenged social identities and the stereotypes held of others^4^. The widespread impact and uncontrollable nature of this event are inconsistent with pre-COVID19 views that attribute ill-intent to those receiving to unemployment benefits (Fiske et al., 2002; Baumberg, 2012; Contini and Richiardi, 2012; Bye et al., 2014). We suggest the changing context altered perceptions of the “deservingness” of people who are unemployed as unemployment in the context of COVID19 is less indicative personal failings or a result of one’s “own doing” (Petersen et al., 2011; van Oorschot and Roosma, 2017). It is important to recognize, however, that the negative perceptions of unemployed benefit recipients were attenuated in the COVID19 assessment, but they continued to be rated less favorably than those who were employed on the key outcome measures.

In contrast to our findings on the key measures of employability and Conscientiousness, the previous and current research is less conclusive for the other outcome measures. The current study showed a broadly consistent gradient in the perception of employed and unemployed characters for all outcome measures apart from Openness and Extraversion. Findings on these other measures have been weaker and inconsistent across previous studies (Schofield and Butterworth, 2018b; Schofield et al., 2019), and the current experiment was not designed with sufficient power to demonstrate interaction effects for these measures. There was, however, one measure that showed significant divergence from the expected profile of results. A significant interaction term suggested that study participants at the Time 2 (COVID19) assessment reported that the employed characters should feel greater levels of Guilt and Shame than those who participated in the pre-COVID19 assessment. In contrast, there was consistency in the ratings of unemployed characters on this measure across the two assessment occasions. While not predicted, these results are also interpretable in the context of the pervasive job loss that accompanied the COVID19 crisis. Haller et al. (2020), for example, argue that the highly distressing, morally difficult, and cumulative nature of COVID19 related stressors presents a perfect storm to result in a guilt and shame responses. The context of mass job losses may leave “surviving” workers feeling increasingly guilty.

The main findings of the current study are consistent with previous experimental studies that show that the stereotypes of unemployed benefit recipients are malleable (Aarøe, 2011; Schofield et al., 2019). These previous studies, however, have demonstrated malleability by providing additional information about unemployed individuals that was inconsistent with the unemployed benefit recipient stereotype (e.g., the external causes of their unemployment). In contrast, the current study did not change how the vignette characters were presented or the experimental procedures. Rather, we assessed how the changing context in which study participants were living had altered their perceptions: suggesting the experience of COVID19 altered stereotypical views held by study participants rather than presenting information about the character that would challenge the applicability of the benefit recipient stereotype in this instance.

Perceptions and stereotypes of benefit recipients can be reinforced (and potentially generated) by government actions and policies. Structural stigma can be used as a policy tool to stigmatize benefit receipt as a strategy to reduce dependence on income support and encourage workforce participation (Moffitt, 1983; Stuber and Schlesinger, 2006; Baumberg, 2012; Contini and Richiardi, 2012; Garthwaite, 2013). In the current instance, however, the Australian government acted quickly to provide greater support to Australians who lost their jobs (e.g., doubling the rate of payment, removing mandatory reporting to the welfare services) and this may have reduced the stigmatizing structural features of the income support system and contributed to the changed perceptions of benefit recipients identified in this study.

### Limitations

The current study took advantage of a natural experimental design and replicated a pre-COVID19 study during the COVID19 crisis. The study is limited by the relatively small sample size at Time 1, which was not designed for current purposes but part of another study. We were not able to include most of the participants from the original Time 1 study as most of the experimental conditions described activities that were illegal/inconsistent with recommend activity at the time of the COVID19 lockdown and social restriction measures. Finally, the data collection for the current study occurred very quickly after the initial and sudden COVID19 lockdowns and economic shock, which is both a strength and a limitation for the generalizability of the results. The pattern of results using the same sampling frame offers compelling support for our hypothesis that the shared economic shock and increase in unemployment attenuates stigmatizing community attitudes toward those who need to receive benefits. Our current conclusions would be further strengthened by a subsequent replication when the public health and economic crises stabilize, to test whether pre-COVID perceptions return.

## Conclusion

The current study provides novel information about impact of the COVID19 health and economic crisis, and the impact of the corresponding policy responses on community perceptions. This novel study shows how community perceptions of employment and benefit recipient status have been altered by the COVID19 pandemic. These results add to knowledge about the determinants of welfare stigma, particularly relating to employability, highlighting societal level contextual factors.

## Data Availability Statement

The raw data supporting the conclusions of this article will be made available by the authors, without undue reservation.

## Ethics Statement

The studies involving human participants were reviewed and approved by Melbourne University Human Research Ethics Committee. The patients/participants provided their written informed consent to participate in this study.

## Author Contributions

AS led the review conceptualized by TS and PB. AS and PB conducted the analyses and wrote up the review. TS led the data collection, reviewed and edited the manuscript, and provided data management support. This manuscript is based on previous extensive work by TS and PB on stereotypes toward the unemployed and welfare benefit recipients. All authors contributed to the article and approved the submitted version.

## Conflict of Interest

The authors declare that the research was conducted in the absence of any commercial or financial relationships that could be construed as a potential conflict of interest.

## Footnotes

1 https://fred.stlouisfed.org/series/UNRATE

2 The Australian figure includes individuals working zero hours who had “no work, not enough work available or were stood down.” The US Bureau of Labor Statistics noted that some people on temporary layoff were not classified as such and the unemployment rate could have been almost 5 percentage points higher.

3 https://osf.io/wknb6

4 https://pursuit.unimelb.edu.au/articles/our-changing-identities-under-covid-19
