# Supplementary material for: Unemployment, Employability and COVID19: How the Global Socioeconomic Shock Challenged Negative Perceptions Toward the Less Fortunate in the Australian Context
Source: Front Psychol. 2020 Oct 15;11:594837. doi: 10.3389/fpsyg.2020.594837 (PMC7593239; doi:10.3389/fpsyg.2020.594837)
Supplement: Supplementary file 1 [file Table_1.DOCX]

Table S1

Adjusted Model for Fixed Effects Estimates of Outcomes as a Function of Interactions between Condition (employed/unemployed) and Time (pre-COVID/COVID)(significant effects (ps<.05) are in **Bold**)

|  | b | SE | z | p | 95%CI | |
| --- | --- | --- | --- | --- | --- | --- |
| Time (ref Time 1, pre-COVID19)) | |  |  |  |  |  |
| Time 2 COVID19 | -0.06 | 0.06 | -0.88 | 0.38 | -0.18 | 0.07 |
| Condition (ref E) | |  |  |  |  |  |
| **UE** | **-0.57** | **0.08** | **-7.5** | **<.001** | **-0.72** | **-0.42** |
|  |  |  |  |  |  |  |
| Time x Condition | |  |  |  |  |  |
| **Time 2 UE** | **0.26** | **0.09** | **2.89** | **<.001** | **0.09** | **0.44** |
|  |  |  |  |  |  |  |
| Participant Age (ref 18-35) | | |  |  |  |  |
| 36-55 | 0.08 | 0.05 | 1.57 | 0.12 | -0.02 | 0.19 |
| 56-85 | -0.08 | 0.06 | -1.30 | 0.19 | -0.19 | 0.04 |
|  |  |  |  |  |  |  |
| Participant gender (ref Male | | |  |  |  |  |
| **Female** | **0.13** | **0.04** | **2.96** | **<.001** | **0.04** | **0.21** |
| Participant employment (ref Employed) | | | |  |  |  |
| Unemployed | -0.05 | 0.05 | -0.97 | 0.33 | -0.15 | 0.05 |
|  |  |  |  |  |  |  |
| Participant education (ref high school) | | | |  |  |  |
| Vocational (TAFE, etc) | -0.01 | 0.06 | -0.25 | 0.80 | -0.13 | 0.10 |
| University (undergrad) | -0.12 | 0.06 | -1.92 | 0.05 | -0.23 | 0.00 |
| University (postgrad) | -0.06 | 0.07 | -0.94 | 0.35 | -0.19 | 0.07 |

Conditions: E=employed; UE=unemployed;

Table S2: Adjusted Models for the Effect of Condition by Time on 14 outcomes (Condition: E=employed: UE=unemployed; significant effects (*p*s<.05) are in **Bold**)

|  |  | *b* | SE | z | *p* | 95%CI | |
| --- | --- | --- | --- | --- | --- | --- | --- |
| **Agreeableness** | | | | |  |  |  |
|  | Time (ref Time1) | |  |  |  |  |  |
|  | Time 2 | 0.05 | 0.09 | 0.63 | 0.53 | -0.12 | 0.22 |
|  | Condition (ref E) | |  |  |  |  |  |
|  | **UE** | **-0.28** | **0.10** | **-2.77** | **0.01** | **-0.48** | **-0.08** |
|  | Time x Condition | |  |  |  |  |  |
|  | Time 2 UE | -0.06 | 0.12 | -0.52 | 0.60 | -0.31 | 0.18 |
|  | Participant Age (ref 18-35) | | |  |  |  |  |
|  | **36-55** | **0.19** | **0.07** | **2.65** | **0.01** | **0.05** | **0.32** |
|  | **56-85** | **0.19** | **0.08** | **2.47** | **0.01** | **0.04** | **0.33** |
|  | Participant gender (ref Male | | |  |  |  |  |
|  | Female | 0.06 | 0.06 | 1.07 | 0.28 | -0.05 | 0.18 |
|  | Participant employment (ref Employed) | | | |  |  |  |
|  | Unemployed | -0.04 | 0.06 | -0.62 | 0.53 | -0.17 | 0.09 |
|  | Participant education (ref high school) | | | |  |  |  |
|  | Vocational (TAFE, etc) | 0.02 | 0.08 | 0.29 | 0.77 | -0.13 | 0.18 |
|  | University (undergrad) | -0.06 | 0.08 | -0.81 | 0.42 | -0.22 | 0.09 |
|  | University (postgrad) | -0.04 | 0.08 | -0.51 | 0.61 | -0.20 | 0.12 |
| **Conscientiousness** | | |  |  |  |  |  |
|  | Time (ref Time1) | |  |  |  |  |  |
|  | Time 2 | -0.08 | 0.12 | -0.61 | 0.54 | -0.32 | 0.17 |
|  | Condition (ref E) | |  |  |  |  |  |
|  | **UE** | **-0.87** | **0.15** | **-5.93** | **<.001** | **-1.16** | **-0.58** |
|  | Time x Condition | |  |  |  |  |  |
|  | **Time 2 UE** | **0.42** | **0.18** | **2.36** | **0.02** | **0.07** | **0.77** |
|  | Participant Age (ref 18-35) | | |  |  |  |  |
|  | **36-55** | **0.30** | **0.10** | **2.88** | **<.001** | **0.09** | **0.50** |
|  | 56-85 | 0.11 | 0.11 | 0.99 | 0.32 | -0.11 | 0.34 |
|  | Participant gender (ref Male | | |  |  |  |  |
|  | Female | 0.09 | 0.08 | 1.03 | 0.30 | -0.08 | 0.25 |
|  | Participant employment (ref Employed) | | | |  |  |  |
|  | Unemployed | -0.16 | 0.10 | -1.57 | 0.12 | -0.36 | 0.04 |
|  | Participant education (ref high school) | | | |  |  |  |
|  | Vocational (TAFE, etc) | -0.12 | 0.12 | -0.99 | 0.32 | -0.34 | 0.11 |
|  | University (undergrad) | -0.22 | 0.12 | -1.84 | 0.07 | -0.46 | 0.01 |
|  | University (postgrad) | -0.18 | 0.13 | -1.37 | 0.17 | -0.44 | 0.08 |
| **Openness** | | |  |  |  |  |  |
|  | Time (ref Time1) | |  |  |  |  |  |
|  | Time 2 | 0.10 | 0.11 | 0.87 | 0.38 | -0.12 | 0.31 |
|  | Condition (ref E) | |  |  |  |  |  |
|  | UE | -0.03 | 0.13 | -0.22 | 0.83 | -0.27 | 0.22 |
|  | Time x Condition | |  |  |  |  |  |
|  | Time 2 UE | 0.05 | 0.16 | 0.34 | 0.73 | -0.25 | 0.36 |
|  | Participant Age (ref 18-35) | | |  |  |  |  |
|  | **36-55** | **0.20** | **0.09** | **2.20** | **0.03** | **0.02** | **0.37** |
|  | 56-85 | 0.04 | 0.10 | 0.38 | 0.70 | -0.16 | 0.24 |
|  | Participant gender (ref Male | | |  |  |  |  |
|  | Female | 0.12 | 0.08 | 1.61 | 0.11 | -0.03 | 0.27 |
|  | Participant employment (ref Employed) | | | |  |  |  |
|  | Unemployed | 0.06 | 0.09 | 0.71 | 0.48 | -0.11 | 0.24 |
|  | Participant education (ref high school) | | | |  |  |  |
|  | Vocational (TAFE, etc) | 0.07 | 0.10 | 0.67 | 0.50 | -0.13 | 0.27 |
|  | University (undergrad) | -0.04 | 0.11 | -0.40 | 0.69 | -0.26 | 0.17 |
|  | University (postgrad) | 0.04 | 0.11 | 0.39 | 0.69 | -0.17 | 0.26 |
| **Extraversion** | | |  |  |  |  |  |
|  | Time (ref Time1) | |  |  |  |  |  |
|  | Time 2 | -0.04 | 0.12 | -0.34 | 0.73 | -0.27 | 0.19 |
|  | Condition (ref E) | |  |  |  |  |  |
|  | UE | -0.17 | 0.13 | -1.35 | 0.18 | -0.42 | 0.08 |
|  | Time x Condition | |  |  |  |  |  |
|  | Time 2 UE | 0.23 | 0.16 | 1.43 | 0.15 | -0.08 | 0.53 |
|  | Participant Age (ref 18-35) | | |  |  |  |  |
|  | 36-55 | -0.02 | 0.09 | -0.18 | 0.86 | -0.20 | 0.16 |
|  | **56-85** | **-0.25** | **0.09** | **-2.70** | **0.01** | **-0.43** | **-0.07** |
|  | Participant gender (ref Male | | |  |  |  |  |
|  | Female | 0.14 | 0.08 | 1.82 | 0.07 | -0.01 | 0.28 |
|  | Participant employment (ref Employed) | | | |  |  |  |
|  | Unemployed | 0.13 | 0.08 | 1.56 | 0.12 | -0.03 | 0.29 |
|  | Participant education (ref high school) | | | |  |  |  |
|  | Vocational (TAFE, etc) | -0.03 | 0.09 | -0.32 | 0.75 | -0.21 | 0.15 |
|  | University (undergrad) | -0.20 | 0.11 | -1.87 | 0.06 | -0.41 | 0.01 |
|  | University (postgrad) | -0.13 | 0.11 | -1.19 | 0.23 | -0.34 | 0.08 |
| **Emotional Stability** | | | |  |  |  |  |
|  | Time (ref Time1) | |  |  |  |  |  |
|  | Time 2 | -0.08 | 0.11 | -0.76 | 0.45 | -0.30 | 0.13 |
|  | Condition (ref E) | |  |  |  |  |  |
|  | **UE** | **-0.44** | **0.12** | **-3.60** | **<.001** | **-0.69** | **-0.20** |
|  | Time x Condition | |  |  |  |  |  |
|  | Time 2 UE | 0.26 | 0.15 | 1.77 | 0.08 | -0.03 | 0.55 |
|  | Participant Age (ref 18-35) | | |  |  |  |  |
|  | 36-55 | 0.11 | 0.09 | 1.32 | 0.19 | -0.06 | 0.28 |
|  | 56-85 | 0.05 | 0.09 | 0.55 | 0.58 | -0.13 | 0.22 |
|  | Participant gender (ref Male | | |  |  |  |  |
|  | **Female** | **0.17** | **0.07** | **2.46** | **0.01** | **0.03** | **0.31** |
|  | Participant employment (ref Employed) | | | |  |  |  |
|  | **Unemployed** | **-0.19** | **0.08** | **-2.40** | **0.02** | **-0.35** | **-0.03** |
|  | Participant education (ref high school) | | | |  |  |  |
|  | Vocational (TAFE, etc) | 0.06 | 0.10 | 0.63 | 0.53 | -0.13 | 0.25 |
|  | University (undergrad) | -0.06 | 0.10 | -0.55 | 0.58 | -0.26 | 0.15 |
|  | University (postgrad) | -0.07 | 0.10 | -0.65 | 0.51 | -0.27 | 0.13 |
| **Worker suitability** | | |  |  |  |  |  |
|  | Time (ref Time1) | |  |  |  |  |  |
|  | Time 2 | -0.17 | 0.14 | -1.26 | 0.21 | -0.44 | 0.10 |
|  | Condition (ref E) | |  |  |  |  |  |
|  | **UE** | **-1.46** | **0.17** | **-8.59** | **<.001** | **-1.80** | **-1.13** |
|  | Time x Condition | |  |  |  |  |  |
|  | **Time 2 UE** | **0.63** | **0.21** | **2.98** | **<.001** | **0.21** | **1.04** |
|  | Participant Age (ref 18-35) | | |  |  |  |  |
|  | 36-55 | 0.05 | 0.12 | 0.38 | 0.70 | -0.19 | 0.29 |
|  | 56-85 | 0.00 | 0.14 | -0.02 | 0.98 | -0.27 | 0.26 |
|  | Participant gender (ref Male | | |  |  |  |  |
|  | Female | 0.10 | 0.10 | 1.00 | 0.32 | -0.10 | 0.30 |
|  | Participant employment (ref Employed) | | | |  |  |  |
|  | Unemployed | -0.11 | 0.12 | -0.92 | 0.36 | -0.35 | 0.13 |
|  | Participant education (ref high school) | | | |  |  |  |
|  | Vocational (TAFE, etc) | 0.14 | 0.14 | 0.99 | 0.32 | -0.14 | 0.41 |
|  | University (undergrad) | -0.14 | 0.14 | -0.98 | 0.33 | -0.41 | 0.14 |
|  | University (postgrad) | -0.01 | 0.15 | -0.08 | 0.94 | -0.31 | 0.29 |
| **Boss suitability** | | |  |  |  |  |  |
|  | Time (ref Time1) | |  |  |  |  |  |
|  | Time 2 | -0.14 | 0.15 | -0.93 | 0.35 | -0.43 | 0.15 |
|  | Condition (ref E) | |  |  |  |  |  |
|  | **UE** | **-1.09** | **0.17** | **-6.55** | **<.001** | **-1.42** | **-0.76** |
|  | Time x Condition | |  |  |  |  |  |
|  | **Time 2 UE** | **0.70** | **0.21** | **3.37** | **<.001** | **0.29** | **1.11** |
|  | Participant Age (ref 18-35) | | |  |  |  |  |
|  | 36-55 | -0.17 | 0.12 | -1.40 | 0.16 | -0.40 | 0.07 |
|  | **56-85** | **-0.65** | **0.13** | **-4.81** | **<.001** | **-0.91** | **-0.38** |
|  | Participant gender (ref Male | | |  |  |  |  |
|  | **Female** | **0.21** | **0.10** | **2.05** | **0.04** | **0.01** | **0.41** |
|  | Participant employment (ref Employed) | | | |  |  |  |
|  | Unemployed | 0.06 | 0.12 | 0.47 | 0.64 | -0.18 | 0.29 |
|  | Participant education (ref high school) | | | |  |  |  |
|  | Vocational (TAFE, etc) | 0.15 | 0.13 | 1.11 | 0.27 | -0.12 | 0.41 |
|  | University (undergrad) | -0.03 | 0.14 | -0.22 | 0.82 | -0.31 | 0.24 |
|  | University (postgrad) | 0.10 | 0.15 | 0.69 | 0.49 | -0.19 | 0.40 |
|  | **Dehumanization** | |  |  |  |  |  |
|  | Time (ref Time1) | |  |  |  |  |  |
|  | Time 2 | 0.01 | 0.08 | 0.12 | 0.91 | -0.14 | 0.16 |
|  | Condition (ref E) | |  |  |  |  |  |
|  | **UE** | **-0.36** | **0.09** | **-3.92** | **<.001** | **-0.55** | **-0.18** |
|  | Time x Condition | |  |  |  |  |  |
|  | Time 2 UE | 0.21 | 0.12 | 1.82 | 0.07 | -0.02 | 0.44 |
|  | Participant Age (ref 18-35) | | |  |  |  |  |
|  | 36-55 | 0.13 | 0.07 | 2.00 | 0.05 | 0.00 | 0.26 |
|  | 56-85 | -0.03 | 0.08 | -0.39 | 0.70 | -0.18 | 0.12 |
|  | Participant gender (ref Male | | |  |  |  |  |
|  | **Female** | **0.13** | **0.06** | **2.26** | **0.02** | **0.02** | **0.24** |
|  | Participant employment (ref Employed) | | | |  |  |  |
|  | Unemployed | -0.07 | 0.07 | -1.03 | 0.30 | -0.20 | 0.06 |
|  | Participant education (ref high school) | | | |  |  |  |
|  | Vocational (TAFE, etc) | 0.03 | 0.08 | 0.39 | 0.70 | -0.12 | 0.18 |
|  | University (undergrad) | -0.06 | 0.08 | -0.75 | 0.46 | -0.22 | 0.10 |
|  | University (postgrad) | -0.03 | 0.08 | -0.37 | 0.71 | -0.19 | 0.13 |
| **Anger & Disgust** | | |  |  |  |  |  |
|  | Time (ref Time1) | |  |  |  |  |  |
|  | Time 2 | 0.02 | 0.11 | 0.16 | 0.87 | -0.21 | 0.24 |
|  | Condition (ref E) | |  |  |  |  |  |
|  | **UE** | **-0.64** | **0.13** | **-4.78** | **<.001** | **-0.90** | **-0.38** |
|  | Time x Condition | |  |  |  |  |  |
|  | Time 2 UE | 0.27 | 0.17 | 1.61 | 0.11 | -0.06 | 0.60 |
|  | Participant Age (ref 18-35) | | |  |  |  |  |
|  | **36-55** | **0.22** | **0.10** | **2.13** | **0.03** | **0.02** | **0.42** |
|  | 56-85 | 0.01 | 0.11 | 0.08 | 0.94 | -0.20 | 0.22 |
|  | Participant gender (ref Male | | |  |  |  |  |
|  | Female | 0.12 | 0.08 | 1.49 | 0.14 | -0.04 | 0.28 |
|  | Participant employment (ref Employed) | | | |  |  |  |
|  | Unemployed | -0.14 | 0.10 | -1.39 | 0.17 | -0.33 | 0.06 |
|  | Participant education (ref high school) | | | |  |  |  |
|  | Vocational (TAFE, etc) | -0.13 | 0.11 | -1.25 | 0.21 | -0.35 | 0.08 |
|  | **University (undergrad)** | **-0.34** | **0.12** | **-2.85** | **0.00** | **-0.57** | **-0.10** |
|  | University (postgrad) | -0.19 | 0.12 | -1.52 | 0.13 | -0.43 | 0.05 |
| **Guilt & Shame** | | |  |  |  |  |  |
|  | Time (ref Time1) | |  |  |  |  |  |
|  | **Time 2** | **-0.55** | **0.13** | **-4.30** | **<.001** | **-0.81** | **-0.30** |
|  | Condition (ref E) | |  |  |  |  |  |
|  | **UE** | **-0.84** | **0.15** | **-5.48** | **<.001** | **-1.14** | **-0.54** |
|  | Time x Condition | |  |  |  |  |  |
|  | **Time 2 UE** | **0.67** | **0.19** | **3.62** | **<.001** | **0.31** | **1.04** |
|  | Participant Age (ref 18-35) | | |  |  |  |  |
|  | 36-55 | 0.17 | 0.11 | 1.51 | 0.13 | -0.05 | 0.38 |
|  | 56-85 | 0.02 | 0.11 | 0.15 | 0.88 | -0.21 | 0.24 |
|  | Participant gender (ref Male | | |  |  |  |  |
|  | Female | 0.10 | 0.09 | 1.16 | 0.25 | -0.07 | 0.28 |
|  | Participant employment (ref Employed) | | | |  |  |  |
|  | Unemployed | -0.04 | 0.11 | -0.36 | 0.72 | -0.25 | 0.17 |
|  | Participant education (ref high school) | | | |  |  |  |
|  | Vocational (TAFE, etc) | -0.09 | 0.12 | -0.71 | 0.48 | -0.33 | 0.15 |
|  | University (undergrad) | -0.10 | 0.13 | -0.77 | 0.44 | -0.34 | 0.15 |
|  | University (postgrad) | 0.00 | 0.13 | -0.03 | 0.97 | -0.27 | 0.26 |
| **Communion/Morality** | |  |  |  |  |  |  |
|  | Time (ref Time1) | |  |  |  |  |  |
|  | Time 2 | -0.03 | 0.07 | -0.46 | 0.64 | -0.16 | 0.10 |
|  | Condition (ref E) | |  |  |  |  |  |
|  | **UE** | **-0.45** | **0.08** | **-5.54** | **0.00** | **-0.61** | **-0.29** |
|  | Time x Condition | |  |  |  |  |  |
|  | Time 2 UE | 0.18 | 0.10 | 1.81 | 0.07 | -0.02 | 0.38 |
|  | Participant Age (ref 18-35) | | |  |  |  |  |
|  | 36-55 | 0.04 | 0.06 | 0.74 | 0.46 | -0.07 | 0.16 |
|  | 56-85 | 0.02 | 0.07 | 0.28 | 0.78 | -0.11 | 0.15 |
|  | Participant gender (ref Male | | |  |  |  |  |
|  | Female | 0.04 | 0.05 | 0.88 | 0.38 | -0.05 | 0.14 |
|  | Participant employment (ref Employed) | | | |  |  |  |
|  | Unemployed | -0.03 | 0.06 | -0.43 | 0.67 | -0.14 | 0.09 |
|  | Participant education (ref high school) | | | |  |  |  |
|  | Vocational (TAFE, etc) | 0.00 | 0.07 | 0.07 | 0.95 | -0.13 | 0.14 |
|  | University (undergrad) | -0.04 | 0.07 | -0.49 | 0.63 | -0.18 | 0.11 |
|  | University (postgrad) | -0.03 | 0.08 | -0.35 | 0.73 | -0.18 | 0.12 |
| **Agency/assertiveness** | | |  |  |  |  |  |
|  | Time (ref Time1) | |  |  |  |  |  |
|  | Time 2 | -0.04 | 0.07 | -0.63 | 0.53 | -0.17 | 0.09 |
|  | Condition (ref E) | |  |  |  |  |  |
|  | **UE** | **-0.46** | **0.08** | **-5.73** | **<.001** | **-0.62** | **-0.30** |
|  | Time x Condition | |  |  |  |  |  |
|  | Time 2 UE | 0.16 | 0.10 | 1.53 | 0.13 | -0.05 | 0.36 |
|  | Participant Age (ref 18-35) | | |  |  |  |  |
|  | 36-55 | 0.06 | 0.06 | 1.03 | 0.30 | -0.06 | 0.19 |
|  | 56-85 | -0.02 | 0.07 | -0.26 | 0.79 | -0.15 | 0.12 |
|  | Participant gender (ref Male | | |  |  |  |  |
|  | Female | 0.03 | 0.05 | 0.50 | 0.62 | -0.07 | 0.12 |
|  | Participant employment (ref Employed) | | | |  |  |  |
|  | Unemployed | -0.01 | 0.06 | -0.10 | 0.92 | -0.12 | 0.11 |
|  | Participant education (ref high school) | | | |  |  |  |
|  | Vocational (TAFE, etc) | 0.02 | 0.07 | 0.30 | 0.77 | -0.12 | 0.17 |
|  | University (undergrad) | -0.02 | 0.08 | -0.20 | 0.85 | -0.17 | 0.14 |
|  | University (postgrad) | 0.00 | 0.09 | -0.01 | 0.99 | -0.17 | 0.17 |
| **Agency/competence** | | |  |  |  |  |  |
|  | Time (ref Time1) | |  |  |  |  |  |
|  | Time 2 | -0.06 | 0.07 | -0.86 | 0.39 | -0.20 | 0.08 |
|  | Condition (ref E) | |  |  |  |  |  |
|  | **UE** | **-0.39** | **0.09** | **-4.46** | **<.001** | **-0.55** | **-0.22** |
|  | Time x Condition | |  |  |  |  |  |
|  | Time 2 UE | 0.19 | 0.11 | 1.71 | 0.09 | -0.03 | 0.40 |
|  | Participant Age (ref 18-35) | | |  |  |  |  |
|  | **36-55** | **0.18** | **0.07** | **2.68** | **0.01** | **0.05** | **0.31** |
|  | 56-85 | 0.08 | 0.07 | 1.04 | 0.30 | -0.07 | 0.22 |
|  | Participant gender (ref Male | | |  |  |  |  |
|  | Female | 0.10 | 0.05 | 1.86 | 0.06 | -0.01 | 0.21 |
|  | Participant employment (ref Employed) | | | |  |  |  |
|  | Unemployed | -0.09 | 0.07 | -1.28 | 0.20 | -0.23 | 0.05 |
|  | Participant education (ref high school) | | | |  |  |  |
|  | Vocational (TAFE, etc) | 0.03 | 0.08 | 0.43 | 0.66 | -0.12 | 0.19 |
|  | University (undergrad) | 0.00 | 0.08 | -0.04 | 0.97 | -0.16 | 0.16 |
|  | University (postgrad) | -0.03 | 0.09 | -0.30 | 0.77 | -0.19 | 0.14 |
| **Communion/Warmth** | |  |  |  |  |  |  |
|  | Time (ref Time1) |  |  |  |  |  |  |
|  | Time 2 | -0.07 | 0.07 | -1.01 | 0.31 | -0.20 | 0.07 |
|  | Condition (ref E) | |  |  |  |  |  |
|  | **UE** | **-0.40** | **0.08** | **-5.01** | **<.001** | **-0.56** | **-0.24** |
|  | Time x Condition |  |  |  |  |  |  |
|  | Time 2 UE | 0.17 | 0.10 | 1.72 | 0.09 | -0.02 | 0.37 |
|  | Participant Age (ref 18-35) | | |  |  |  |  |
|  | 36-55 | -0.02 | 0.06 | -0.30 | 0.76 | -0.13 | 0.10 |
|  | **56-85** | **-0.16** | **0.06** | **-2.48** | **0.01** | **-0.28** | **-0.03** |
|  | Participant gender (ref Male) | | |  |  |  |  |
|  | Female | 0.09 | 0.05 | 1.84 | 0.07 | -0.01 | 0.18 |
|  | Participant employment (ref Employed) | | |  |  |  |  |
|  | Unemployed | 0.00 | 0.06 | -0.05 | 0.96 | -0.11 | 0.11 |
|  | Participant education (ref high school) | | |  |  |  |  |
|  | Vocational (TAFE, etc) | 0.02 | 0.07 | 0.32 | 0.75 | -0.11 | 0.15 |
|  | University (undergrad) | 0.05 | 0.07 | -0.67 | 0.50 | -0.19 | 0.09 |
|  | University (postgrad) | -0.02 | 0.07 | -0.20 | 0.84 | -0.16 | 0.13 |

Table S3: Adjusted Models for the Effect of Condition by Time on 14 outcomes (Condition: E=employed: WP=working poor; UE=unemployed; UB=unemployed on benefits)

| **OUTCOME** | Factor/covariate | *b* | SE | z | *p* | 95% CI | |
| --- | --- | --- | --- | --- | --- | --- | --- |
| **Agreeableness** | |  |  |  |  |  |  |
|  | Time (ref Time 1) | |  |  |  |  |  |
|  | Time 2 | 0.00 | 0.12 | 0.03 | 0.97 | -0.24 | 0.25 |
|  |  |  |  |  |  |  |  |
|  | Condition (ref E) | |  |  |  |  |  |
|  | WP | -0.04 | 0.15 | -0.30 | 0.76 | -0.34 | 0.25 |
|  | UE | -0.26 | 0.15 | -1.71 | 0.09 | -0.55 | 0.04 |
|  | UB | **-0.35** | **0.14** | **-2.46** | **0.01** | **-0.64** | **-0.07** |
|  | Time X Condition | |  |  |  |  |  |
|  | Time 2 WP | 0.10 | 0.17 | 0.57 | 0.57 | -0.24 | 0.43 |
|  | Time 2 UE | -0.06 | 0.18 | -0.37 | 0.71 | -0.41 | 0.28 |
|  | Time 2 UB | 0.04 | 0.18 | 0.22 | 0.83 | -0.31 | 0.39 |
|  | Participant age (ref 18-35yo) | | |  |  |  |  |
|  | **36-55yo** | **0.18** | **0.07** | **2.58** | **0.01** | **0.04** | **0.32** |
|  | **56-85yo** | **0.18** | **0.08** | **2.37** | **0.02** | **0.03** | **0.33** |
|  | Participant gender (ref male) | | |  |  |  |  |
|  | female | 0.06 | 0.06 | 1.05 | 0.30 | -0.05 | 0.17 |
|  | Participant employment (ref unemployed) | | | | |  |  |
|  | employed | -0.05 | 0.06 | -0.73 | 0.47 | -0.17 | 0.08 |
|  | Highest level of participant educ. (ref High school) | | | | |  |  |
|  | Vocational (TAFE, etc) | 0.02 | 0.08 | 0.24 | 0.81 | -0.13 | 0.17 |
|  | Univ. (undergrad) | -0.06 | 0.08 | -0.81 | 0.42 | -0.22 | 0.09 |
|  | Univ. (postgrad) | -0.04 | 0.08 | -0.53 | 0.60 | -0.21 | 0.12 |
| **Conscientiousness** | |  |  |  |  |  |  |
|  | Time (ref Time 1) | |  |  |  |  |  |
|  | Time 2 | -0.11 | 0.16 | -0.65 | 0.52 | -0.43 | 0.22 |
|  | Condition (ref E) | |  |  |  |  |  |
|  | WP | -0.03 | 0.21 | -0.13 | 0.90 | -0.44 | 0.39 |
|  | **UE** | **-0.74** | **0.20** | **-3.75** | **0.00** | **-1.12** | **-0.35** |
|  | **UB** | **-1.03** | **0.20** | **-5.26** | **0.00** | **-1.42** | **-0.65** |
|  | Time X Condition | |  |  |  |  |  |
|  | Time 2 WP | 0.06 | 0.25 | 0.23 | 0.82 | -0.43 | 0.54 |
|  | Time 2 UE | 0.35 | 0.24 | 1.45 | 0.15 | -0.12 | 0.83 |
|  | **Time 2 UB** | **0.55** | **0.25** | **2.21** | **0.03** | **0.06** | **1.03** |
|  | Participant age (ref 18-35yo) | | |  |  |  |  |
|  | **36-55yo** | **0.29** | **0.10** | **2.85** | **0.01** | **0.09** | **0.49** |
|  | 56-85yo | 0.10 | 0.12 | 0.89 | 0.37 | -0.13 | 0.33 |
|  | Participant gender (ref male) | | |  |  |  |  |
|  | female | 0.09 | 0.08 | 1.03 | 0.30 | -0.08 | 0.25 |
|  | Participant employment (ref unemployed) | | | | |  |  |
|  | employed | -0.17 | 0.10 | -1.69 | 0.09 | -0.37 | 0.03 |
|  | Highest level of participant educ. (ref High school) | | | | |  |  |
|  | Vocational (TAFE, etc) | -0.12 | 0.12 | -1.00 | 0.32 | -0.35 | 0.11 |
|  | Univ. (undergrad) | -0.21 | 0.12 | -1.77 | 0.08 | -0.45 | 0.02 |
|  | Univ. (postgrad) | -0.18 | 0.13 | -1.35 | 0.18 | -0.45 | 0.08 |
| **Openness** | |  |  |  |  |  |  |
|  | Time (ref Time 1) | |  |  |  |  |  |
|  | Time 2 | 0.16 | 0.15 | 1.06 | 0.29 | -0.14 | 0.47 |
|  | Condition (ref E) | |  |  |  |  |  |
|  | WP | -0.03 | 0.18 | -0.14 | 0.89 | -0.38 | 0.32 |
|  | UE | 0.03 | 0.16 | 0.16 | 0.88 | -0.30 | 0.35 |
|  | UB | -0.11 | 0.18 | -0.58 | 0.56 | -0.46 | 0.25 |
|  | Time X Condition | |  |  |  |  |  |
|  | Time 2 WP | -0.13 | 0.22 | -0.58 | 0.56 | -0.55 | 0.30 |
|  | Time 2 UE | -0.03 | 0.21 | -0.12 | 0.90 | -0.44 | 0.39 |
|  | Time 2 UB | 0.00 | 0.23 | -0.01 | 0.99 | -0.46 | 0.46 |
|  | Participant age (ref 18-35yo) | | |  |  |  |  |
|  | **36-55yo** | **0.20** | **0.09** | **2.28** | **0.02** | **0.03** | **0.38** |
|  | 56-85yo | 0.03 | 0.10 | 0.33 | 0.74 | -0.17 | 0.23 |
|  | Participant gender (ref male) | | |  |  |  |  |
|  | female | 0.12 | 0.08 | 1.65 | 0.10 | -0.02 | 0.27 |
|  | Participant employment (ref unemployed) | | | | |  |  |
|  | employed | 0.06 | 0.09 | 0.65 | 0.52 | -0.12 | 0.24 |
|  | Highest level of participant educ. (ref High school) | | | | |  |  |
|  | Vocational (TAFE, etc) | 0.08 | 0.10 | 0.75 | 0.45 | -0.13 | 0.28 |
|  | Univ. (undergrad) | -0.03 | 0.11 | -0.32 | 0.75 | -0.25 | 0.18 |
|  | Univ. (postgrad) | 0.05 | 0.11 | 0.44 | 0.66 | -0.17 | 0.26 |
| **Extraversion** | |  |  |  |  |  |  |
|  | Time (ref Time 1) | |  |  |  |  |  |
|  | Time 2 | 0.00 | 0.17 | 0.00 | 1.00 | -0.33 | 0.32 |
|  | Condition (ref E) | |  |  |  |  |  |
|  | WP | -0.16 | 0.20 | -0.82 | 0.41 | -0.55 | 0.23 |
|  | UE | -0.23 | 0.18 | -1.29 | 0.20 | -0.59 | 0.12 |
|  | UB | -0.27 | 0.18 | -1.51 | 0.13 | -0.63 | 0.08 |
|  | Time X Condition | |  |  |  |  |  |
|  | Time 2 WP | -0.07 | 0.23 | -0.29 | 0.78 | -0.52 | 0.39 |
|  | Time 2 UE | 0.06 | 0.22 | 0.28 | 0.78 | -0.38 | 0.50 |
|  | Time 2 UB | 0.31 | 0.22 | 1.42 | 0.16 | -0.12 | 0.75 |
|  | Participant age (ref 18-35yo) | | |  |  |  |  |
|  | 36-55yo | -0.01 | 0.09 | -0.11 | 0.92 | -0.19 | 0.17 |
|  | **56-85yo** | **-0.26** | **0.09** | **-2.81** | **0.01** | **-0.44** | **-0.08** |
|  | Participant gender (ref male) | | |  |  |  |  |
|  | female | 0.13 | 0.08 | 1.77 | 0.08 | -0.01 | 0.28 |
|  | Participant employment (ref unemployed) | | | | |  |  |
|  | employed | 0.13 | 0.08 | 1.51 | 0.13 | -0.04 | 0.29 |
|  | Highest level of participant educ. (ref High school) | | | | |  |  |
|  | Vocational (TAFE, etc) | -0.02 | 0.09 | -0.27 | 0.79 | -0.21 | 0.16 |
|  | Univ. (undergrad) | -0.20 | 0.11 | -1.90 | 0.06 | -0.41 | 0.01 |
|  | Univ. (postgrad) | -0.14 | 0.11 | -1.27 | 0.20 | -0.35 | 0.08 |
| **Emotional Stability** | |  |  |  |  |  |  |
|  | Time (ref Time 1) | |  |  |  |  |  |
|  | Time 2 | -0.10 | 0.15 | -0.62 | 0.53 | -0.40 | 0.21 |
|  | Condition (ref E) | |  |  |  |  |  |
|  | WP | 0.18 | 0.18 | 1.00 | 0.32 | -0.18 | 0.54 |
|  | UE | -0.24 | 0.17 | -1.44 | 0.15 | -0.57 | 0.09 |
|  | **UB** | **-0.47** | **0.19** | **-2.50** | **0.01** | **-0.84** | **-0.10** |
|  | Time X Condition | |  |  |  |  |  |
|  | Time 2 WP | 0.01 | 0.22 | 0.06 | 0.95 | -0.41 | 0.44 |
|  | Time 2 UE | 0.16 | 0.20 | 0.80 | 0.43 | -0.23 | 0.55 |
|  | Time 2 UB | 0.40 | 0.22 | 1.81 | 0.07 | -0.03 | 0.83 |
|  | Participant age (ref 18-35yo) | | |  |  |  |  |
|  | 36-55yo | 0.11 | 0.09 | 1.23 | 0.22 | -0.06 | 0.28 |
|  | 56-85yo | 0.05 | 0.09 | 0.60 | 0.55 | -0.12 | 0.23 |
|  | Participant gender (ref male) | | |  |  |  |  |
|  | **female** | **0.17** | **0.07** | **2.43** | **0.02** | **0.03** | **0.31** |
|  | Participant employment (ref unemployed) | | | | |  |  |
|  | **employed** | **-0.19** | **0.08** | **-2.41** | **0.02** | **-0.35** | **-0.04** |
|  | Highest level of participant educ. (ref High school) | | | | |  |  |
|  | Vocational (TAFE, etc) | 0.05 | 0.10 | 0.52 | 0.60 | -0.14 | 0.24 |
|  | Univ. (undergrad) | -0.06 | 0.10 | -0.53 | 0.59 | -0.26 | 0.15 |
|  | Univ. (postgrad) | -0.08 | 0.10 | -0.73 | 0.47 | -0.28 | 0.13 |
| **Worker suitability** | |  |  |  |  |  |  |
|  | Time (ref Time 1) | |  |  |  |  |  |
|  | Time 2 | -0.27 | 0.19 | -1.43 | 0.15 | -0.65 | 0.10 |
|  | Condition (ref E) | |  |  |  |  |  |
|  | WP | -0.13 | 0.22 | -0.59 | 0.55 | -0.56 | 0.30 |
|  | **UE** | **-1.45** | **0.24** | **-6.18** | **0.00** | **-1.92** | **-0.99** |
|  | **UB** | **-1.61** | **0.24** | **-6.78** | **0.00** | **-2.07** | **-1.14** |
|  | Time X Condition | |  |  |  |  |  |
|  | Time 2 WP | 0.20 | 0.27 | 0.75 | 0.45 | -0.32 | 0.73 |
|  | **Time 2 UE** | **0.63** | **0.29** | **2.17** | **0.03** | **0.06** | **1.21** |
|  | **Time 2 UB** | **0.83** | **0.30** | **2.73** | **0.01** | **0.23** | **1.42** |
|  | Participant age (ref 18-35yo) | | |  |  |  |  |
|  | 36-55yo | 0.04 | 0.12 | 0.32 | 0.75 | -0.20 | 0.28 |
|  | 56-85yo | -0.02 | 0.14 | -0.12 | 0.91 | -0.28 | 0.25 |
|  | Participant gender (ref male) | | |  |  |  |  |
|  | female | 0.10 | 0.10 | 0.97 | 0.33 | -0.10 | 0.30 |
|  | Participant employment (ref unemployed) | | | | |  |  |
|  | employed | -0.13 | 0.12 | -1.01 | 0.31 | -0.37 | 0.12 |
|  | Highest level of participant educ. (ref High school) | | | | |  |  |
|  | Vocational (TAFE, etc) | 0.13 | 0.14 | 0.94 | 0.35 | -0.14 | 0.41 |
|  | Univ. (undergrad) | -0.14 | 0.14 | -0.99 | 0.33 | -0.42 | 0.14 |
|  | Univ. (postgrad) | -0.02 | 0.15 | -0.11 | 0.92 | -0.31 | 0.28 |
| **Boss suitability** | |  |  |  |  |  |  |
|  | Time (ref Time 1) | |  |  |  |  |  |
|  | Time 2 | -0.22 | 0.21 | -1.04 | 0.30 | -0.63 | 0.19 |
|  | Condition (ref E) | |  |  |  |  |  |
|  | WP | -0.12 | 0.24 | -0.48 | 0.63 | -0.59 | 0.36 |
|  | **UE** | **-1.19** | **0.23** | **-5.20** | **0.00** | **-1.64** | **-0.74** |
|  | **UB** | **-1.10** | **0.23** | **-4.72** | **0.00** | **-1.56** | **-0.64** |
|  | Time X Condition | |  |  |  |  |  |
|  | Time 2 WP | 0.16 | 0.29 | 0.55 | 0.58 | -0.41 | 0.73 |
|  | **Time 2 UE** | **0.76** | **0.29** | **2.62** | **0.01** | **0.19** | **1.33** |
|  | **Time 2 UB** | **0.80** | **0.30** | **2.68** | **0.01** | **0.21** | **1.39** |
|  | Participant age (ref 18-35yo) | | |  |  |  |  |
|  | 36-55yo | -0.17 | 0.12 | -1.43 | 0.15 | -0.41 | 0.06 |
|  | **56-85yo** | **-0.65** | **0.14** | **-4.77** | **0.00** | **-0.92** | **-0.38** |
|  | Participant gender (ref male) | | |  |  |  |  |
|  | **female** | **0.20** | **0.10** | **2.02** | **0.04** | **0.01** | **0.40** |
|  | Participant employment (ref unemployed) | | | | |  |  |
|  | employed | 0.05 | 0.12 | 0.44 | 0.66 | -0.18 | 0.29 |
|  | Highest level of participant educ. (ref High school) | | | | |  |  |
|  | Vocational (TAFE, etc) | 0.14 | 0.13 | 1.07 | 0.28 | -0.12 | 0.41 |
|  | Univ. (undergrad) | -0.04 | 0.14 | -0.28 | 0.78 | -0.32 | 0.24 |
|  | Univ. (postgrad) | 0.10 | 0.15 | 0.65 | 0.51 | -0.20 | 0.40 |
| **Dehumanisation** | |  |  |  |  |  |  |
|  | Time (ref Time 1) | |  |  |  |  |  |
|  | Time 2 | -0.05 | 0.11 | -0.50 | 0.62 | -0.27 | 0.16 |
|  | Condition (ref E) | |  |  |  |  |  |
|  | WP | 0.08 | 0.13 | 0.62 | 0.53 | -0.17 | 0.33 |
|  | **UE** | **-0.32** | **0.14** | **-2.35** | **0.02** | **-0.59** | **-0.05** |
|  | **UB** | **-0.33** | **0.13** | **-2.59** | **0.01** | **-0.59** | **-0.08** |
|  | Time X Condition | |  |  |  |  |  |
|  | Time 2 WP | 0.12 | 0.15 | 0.76 | 0.45 | -0.18 | 0.41 |
|  | Time 2 UE | 0.29 | 0.17 | 1.72 | 0.09 | -0.04 | 0.61 |
|  | Time 2 UB | 0.26 | 0.17 | 1.59 | 0.11 | -0.06 | 0.59 |
|  | Participant age (ref 18-35yo) | | |  |  |  |  |
|  | 36-55yo | 0.12 | 0.07 | 1.90 | 0.06 | 0.00 | 0.25 |
|  | 56-85yo | -0.03 | 0.08 | -0.35 | 0.73 | -0.18 | 0.12 |
|  | Participant gender (ref male) | | |  |  |  |  |
|  | **female** | **0.13** | **0.06** | **2.26** | **0.02** | **0.02** | **0.24** |
|  | Participant employment (ref unemployed) | | | | |  |  |
|  | employed | -0.07 | 0.07 | -1.05 | 0.29 | -0.20 | 0.06 |
|  |  |  |  |  |  |  |  |
|  | Highest level of participant educ. (ref High school) | | | | |  |  |
|  | Vocational (TAFE, etc) | 0.02 | 0.08 | 0.29 | 0.78 | -0.13 | 0.18 |
|  | Univ. (undergrad) | -0.06 | 0.08 | -0.76 | 0.45 | -0.22 | 0.10 |
|  | Univ. (postgrad) | -0.03 | 0.08 | -0.37 | 0.71 | -0.20 | 0.13 |
| **Anger & Disgust** | |  |  |  |  |  |  |
|  | Time (ref Time 1) | |  |  |  |  |  |
|  | Time 2 | -0.04 | 0.17 | -0.25 | 0.80 | -0.37 | 0.29 |
|  |  |  |  |  |  |  |  |
|  | Condition (ref E) | |  |  |  |  |  |
|  | WP | 0.10 | 0.19 | 0.55 | 0.58 | -0.26 | 0.47 |
|  | **UE** | **-0.58** | **0.20** | **-2.94** | **0.00** | **-0.96** | **-0.19** |
|  | **UB** | **-0.60** | **0.19** | **-3.12** | **0.00** | **-0.98** | **-0.22** |
|  |  |  |  |  |  |  |  |
|  | Time X Condition | |  |  |  |  |  |
|  | Time 2 WP | 0.11 | 0.22 | 0.48 | 0.63 | -0.33 | 0.55 |
|  | Time 2 UE | 0.39 | 0.25 | 1.55 | 0.12 | -0.10 | 0.88 |
|  | Time 2 UB | 0.27 | 0.24 | 1.14 | 0.25 | -0.20 | 0.75 |
|  |  |  |  |  |  |  |  |
|  | Participant age (ref 18-35yo) | | |  |  |  |  |
|  | **36-55yo** | **0.21** | **0.10** | **2.07** | **0.04** | **0.01** | **0.41** |
|  | 56-85yo | 0.01 | 0.11 | 0.11 | 0.91 | -0.20 | 0.22 |
|  |  |  |  |  |  |  |  |
|  | Participant gender (ref male) | | |  |  |  |  |
|  | female | 0.12 | 0.08 | 1.50 | 0.13 | -0.04 | 0.29 |
|  | Participant employment (ref unemployed) | | | | |  |  |
|  | employed | -0.14 | 0.10 | -1.39 | 0.16 | -0.33 | 0.06 |
|  |  |  |  |  |  |  |  |
|  | Highest level of participant educ. (ref High school) | | | | |  |  |
|  | Vocational (TAFE, etc) | -0.14 | 0.11 | -1.30 | 0.19 | -0.35 | 0.07 |
|  | **Univ. (undergrad)** | **-0.33** | **0.12** | **-2.85** | **0.00** | **-0.56** | **-0.10** |
|  | Univ. (postgrad) | -0.18 | 0.12 | -1.47 | 0.14 | -0.43 | 0.06 |
| **Guilt & Shame** | |  |  |  |  |  |  |
|  | Time (ref Time 1) | |  |  |  |  |  |
|  | **Time 2** | **-0.54** | **0.19** | **-2.94** | **0.00** | **-0.91** | **-0.18** |
|  | Condition (ref E) | |  |  |  |  |  |
|  | WP | 0.17 | 0.21 | 0.79 | 0.43 | -0.25 | 0.59 |
|  | **UE** | **-0.63** | **0.22** | **-2.85** | **0.01** | **-1.06** | **-0.19** |
|  | **UB** | **-0.89** | **0.22** | **-4.05** | **0.00** | **-1.33** | **-0.46** |
|  | Time X Condition | |  |  |  |  |  |
|  | Time 2 WP | -0.03 | 0.26 | -0.11 | 0.91 | -0.53 | 0.47 |
|  | Time 2 UE | 0.48 | 0.27 | 1.80 | 0.07 | -0.04 | 1.00 |
|  | **Time 2 UB** | **0.86** | **0.26** | **3.25** | **0.00** | **0.34** | **1.37** |
|  | Participant age (ref 18-35yo) | | |  |  |  |  |
|  | 36-55yo | 0.16 | 0.11 | 1.44 | 0.15 | -0.06 | 0.37 |
|  | 56-85yo | 0.02 | 0.12 | 0.18 | 0.86 | -0.21 | 0.25 |
|  | Participant gender (ref male) | | |  |  |  |  |
|  | female | 0.10 | 0.09 | 1.11 | 0.27 | -0.08 | 0.27 |
|  | Participant employment (ref unemployed) | | | | |  |  |
|  | employed | -0.04 | 0.11 | -0.38 | 0.71 | -0.25 | 0.17 |
|  |  |  |  |  |  |  |  |
|  | Highest level of participant educ. (ref High school) | | | | |  |  |
|  | Vocational (TAFE, etc) | -0.10 | 0.12 | -0.81 | 0.42 | -0.34 | 0.14 |
|  | Univ. (undergrad) | -0.10 | 0.13 | -0.78 | 0.44 | -0.34 | 0.15 |
|  | Univ. (postgrad) | -0.02 | 0.13 | -0.14 | 0.89 | -0.28 | 0.24 |
| **Communion/Morality** | | |  |  |  |  |  |
|  | Time (ref Time 1) | |  |  |  |  |  |
|  | Time 2 | 0.01 | 0.08 | 0.17 | 0.87 | -0.15 | 0.18 |
|  | Condition (ref E) | |  |  |  |  |  |
|  | WP | 0.17 | 0.11 | 1.52 | 0.13 | -0.05 | 0.38 |
|  | **UE** | **-0.28** | **0.11** | **-2.51** | **0.01** | **-0.50** | **-0.06** |
|  | **UB** | **-0.46** | **0.11** | **-4.33** | **0.00** | **-0.66** | **-0.25** |
|  |  |  |  |  |  |  |  |
|  | Time X Condition | |  |  |  |  |  |
|  | Time 2 WP | -0.10 | 0.13 | -0.74 | 0.46 | -0.35 | 0.16 |
|  | Time 2 UE | 0.06 | 0.13 | 0.43 | 0.67 | -0.21 | 0.32 |
|  | Time 2 UB | 0.22 | 0.14 | 1.59 | 0.11 | -0.05 | 0.49 |
|  | Participant age (ref 18-35yo) | | |  |  |  |  |
|  | 36-55yo | 0.04 | 0.06 | 0.70 | 0.48 | -0.08 | 0.16 |
|  | 56-85yo | 0.02 | 0.07 | 0.37 | 0.71 | -0.10 | 0.15 |
|  | Participant gender (ref male) | | |  |  |  |  |
|  | female | 0.04 | 0.05 | 0.87 | 0.39 | -0.05 | 0.14 |
|  | Participant employment (ref unemployed) | | | | |  |  |
|  | employed | -0.02 | 0.06 | -0.42 | 0.67 | -0.14 | 0.09 |
|  | Highest level of participant educ. (ref High school) | | | | |  |  |
|  | Vocational (TAFE, etc) | 0.00 | 0.07 | 0.02 | 0.99 | -0.14 | 0.14 |
|  | Univ. (undergrad) | -0.03 | 0.07 | -0.44 | 0.66 | -0.18 | 0.11 |
|  | Univ. (postgrad) | -0.03 | 0.08 | -0.40 | 0.69 | -0.18 | 0.12 |
| **Communion/warmth** | | |  |  |  |  |  |
|  | Time (ref Time 1) | |  |  |  |  |  |
|  | Time 2 | -.04 | .10 | -0.44 | 0.66 | -0.23 | 0.15 |
|  | Condition (ref E) | |  |  |  |  |  |
|  | WP | 0.11 | 0.11 | 1.00 | 0.32 | -0.11 | 0.34 |
|  | **UE** | **-0.28** | **0.12** | **-2.43** | **0.02** | **-0.51** | **-0.05** |
|  | **UB** | **-0.41** | **0.10** | **-3.92** | **0.00** | **-0.62** | **-0.21** |
|  | Time X Condition | |  |  |  |  |  |
|  | Time 2 WP | -0.06 | 0.14 | -0.44 | 0.66 | -0.32 | 0.21 |
|  | Time 2 UE | 0.06 | 0.14 | 0.44 | 0.66 | -0.21 | 0.34 |
|  | Time 2 UB | 0.23 | 0.14 | 1.64 | 0.10 | -0.04 | 0.50 |
|  | Participant age (ref 18-35yo) | | |  |  |  |  |
|  | 36-55yo | -0.02 | 0.06 | -0.33 | 0.74 | -0.14 | 0.10 |
|  | **56-85yo** | **-0.15** | **0.06** | **-2.39** | **0.02** | **-0.28** | **-0.03** |
|  | Participant gender (ref male) | | |  |  |  |  |
|  | female | 0.09 | 0.05 | 1.80 | 0.07 | -0.01 | 0.18 |
|  | Participant employment (ref unemployed) | | | | |  |  |
|  | employed | 0.00 | 0.06 | -0.05 | 0.96 | -0.11 | 0.11 |
|  | Highest level of participant educ. (ref High school) | | | | |  |  |
|  | Vocational (TAFE, etc) | 0.02 | 0.07 | 0.27 | 0.79 | -0.12 | 0.16 |
|  | Univ. (undergrad) | -0.05 | 0.07 | -0.66 | 0.51 | -0.19 | 0.10 |
|  | Univ. (postgrad) | -0.02 | 0.08 | -0.28 | 0.78 | -0.17 | 0.13 |
| **Agency/assertiveness** | | |  |  |  |  |  |
|  | Time (ref Time 1) | |  |  |  |  |  |
|  | Time 2 | -0.05 | 0.09 | -0.59 | 0.55 | -0.22 | 0.12 |
|  | Condition (ref E) | |  |  |  |  |  |
|  | WP | -0.06 | 0.10 | -0.59 | 0.56 | -0.27 | 0.14 |
|  | **UE** | **-0.50** | **0.11** | **-4.54** | **0.00** | **-0.71** | **-0.28** |
|  | **UB** | **-0.48** | **0.11** | **-4.55** | **0.00** | **-0.69** | **-0.27** |
|  | Time X Condition | |  |  |  |  |  |
|  | Time 2 WP | 0.02 | 0.13 | 0.17 | 0.87 | -0.23 | 0.28 |
|  | Time 2 UE | 0.21 | 0.14 | 1.50 | 0.13 | -0.06 | 0.48 |
|  | Time 2 UB | 0.12 | 0.15 | 0.85 | 0.40 | -0.16 | 0.41 |
|  | Participant age (ref 18-35yo) | | |  |  |  |  |
|  | 36-55yo | 0.07 | 0.06 | 1.05 | 0.29 | -0.06 | 0.19 |
|  | 56-85yo | -0.02 | 0.07 | -0.31 | 0.75 | -0.16 | 0.12 |
|  | Participant gender (ref male) | | |  |  |  |  |
|  | female | 0.03 | 0.05 | 0.53 | 0.60 | -0.07 | 0.13 |
|  | Participant employment (ref unemployed) | | | | |  |  |
|  | employed | -0.01 | 0.06 | -0.14 | 0.89 | -0.13 | 0.11 |
|  | Highest level of participant educ. (ref High school) | | | | |  |  |
|  | Vocational (TAFE, etc) | 0.03 | 0.08 | 0.34 | 0.73 | -0.12 | 0.17 |
|  | Univ. (undergrad) | -0.01 | 0.08 | -0.16 | 0.87 | -0.17 | 0.14 |
|  | Univ. (postgrad) | 0.00 | 0.09 | 0.05 | 0.96 | -0.17 | 0.17 |
| **Agency/competence** | |  |  |  |  |  |  |
|  | Time (ref Time 1) | |  |  |  |  |  |
|  | Time 2 | -0.01 | 0.09 | -0.09 | 0.93 | -0.19 | 0.18 |
|  | Condition (ref E) | |  |  |  |  |  |
|  | WP | 0.12 | 0.11 | 1.06 | 0.29 | -0.10 | 0.34 |
|  | **UE** | **-0.31** | **0.12** | **-2.62** | **0.01** | **-0.54** | **-0.08** |
|  | **UB** | **-0.34** | **0.12** | **-2.89** | **0.00** | **-0.58** | **-0.11** |
|  | Time X Condition | |  |  |  |  |  |
|  | Time 2 WP | -0.11 | 0.14 | -0.77 | 0.44 | -0.38 | 0.17 |
|  | Time 2 UE | 0.11 | 0.15 | 0.75 | 0.46 | -0.18 | 0.40 |
|  | Time 2 UB | 0.16 | 0.16 | 1.03 | 0.30 | -0.14 | 0.47 |
|  | Participant age (ref 18-35yo) | | |  |  |  |  |
|  | **36-55yo** | **0.18** | **0.07** | **2.69** | **0.01** | **0.05** | **0.31** |
|  | 56-85yo | 0.09 | 0.08 | 1.13 | 0.26 | -0.06 | 0.23 |
|  | Participant gender (ref male) | | |  |  |  |  |
|  | female | 0.10 | 0.05 | 1.85 | 0.06 | -0.01 | 0.21 |
|  | Participant employment (ref unemployed) | | | | |  |  |
|  | employed | -0.08 | 0.07 | -1.21 | 0.23 | -0.22 | 0.05 |
|  | Highest level of participant educ. (ref high school) | | | | |  |  |
|  | Vocational (TAFE, etc) | 0.03 | 0.08 | 0.42 | 0.67 | -0.12 | 0.19 |
|  | Univ. (undergrad) | 0.00 | 0.08 | -0.03 | 0.97 | -0.16 | 0.16 |
|  | Univ. (postgrad) | -0.03 | 0.09 | -0.33 | 0.75 | -0.20 | 0.14 |

**Excluded vignettes at Time 1 (pre-COVID19)**

Plays soccer with his local club on Saturday afternoons. This weekend his/her team won an important game. After their game each week the team normally goes to the local sports bar for a couple of beers, but s/he doesn’t normally join. This week s/he decided to join them to celebrate the victory and had a really good time. On Sunday s/he enjoyed a sleep-in and went for a walk at the park, before coming home and catching up on house chores. S/he spent the evening watching a superhero movie on TV and got an early night.

On Saturday s/he went into town to have coffee with a friend before going off to buy groceries. As s/he was walking back to his car he saw someone fall and hurt themselves. S/he stayed with them until the ambulance arrived. On Sunday s/he went for a picnic in the park with family. The weather was perfect and s/he had a great time catching up with his/her relatives.
